# BSR1, a Rice Receptor-like Cytoplasmic Kinase, Positively Regulates Defense Responses to Herbivory

**DOI:** 10.3390/ijms241210395

**Published:** 2023-06-20

**Authors:** Yasukazu Kanda, Tomonori Shinya, Satoru Maeda, Kadis Mujiono, Yuko Hojo, Keisuke Tomita, Kazunori Okada, Takashi Kamakura, Ivan Galis, Masaki Mori

**Affiliations:** 1 Institute of Agrobiological Sciences, NARO (NIAS), Tsukuba 305-8634, Japan; satorum212@gmail.com; 2 Department of Applied Biological Science, Graduate School of Science and Technology, Tokyo University of Science, Noda 278-8510, Japan; kamakura@rs.tus.ac.jp; 3 Institute of Plant Science and Resources, Okayama University, Kurashiki 710-0046, Japan; shinyat@okayama-u.ac.jp (T.S.); kmujiono@faperta.unmul.ac.id (K.M.); y-hojo@okayama-u.ac.jp (Y.H.); igalis@okayama-u.ac.jp (I.G.); 4 Faculty of Agriculture, Mulawarman University, Samarinda 75119, Indonesia; 5 Agro-Biotechnology Research Center, Graduate School of Agricultural and Life Sciences, The University of Tokyo, Tokyo 113-8657, Japan; uktomita@toyaku.ac.jp (K.T.); kokada@g.ecc.u-tokyo.ac.jp (K.O.)

**Keywords:** rice, chewing herbivore, damage-associated molecular pattern (DAMP), Pep, receptor-like cytoplasmic kinase (RLCK), diterpenoid phytoalexin (DP), broad-spectrum resistance

## Abstract

Crops experience herbivory by arthropods and microbial infections. In the interaction between plants and chewing herbivores, lepidopteran larval oral secretions (OS) and plant-derived damage-associated molecular patterns (DAMPs) trigger plant defense responses. However, the mechanisms underlying anti-herbivore defense, especially in monocots, have not been elucidated. The receptor-like cytoplasmic kinase Broad-Spectrum Resistance 1 (BSR1) of *Oryza sativa* L. (rice) mediates cytoplasmic defense signaling in response to microbial pathogens and enhances disease resistance when overexpressed. Here, we investigated whether BSR1 contributes to anti-herbivore defense responses. *BSR1* knockout suppressed rice responses triggered by OS from the chewing herbivore *Mythimna loreyi* Duponchel (Lepidoptera: Noctuidae) and peptidic DAMPs OsPeps, including the activation of genes required for biosynthesis of diterpenoid phytoalexins (DPs). *BSR1*-overexpressing rice plants exhibited hyperactivation of DP accumulation and ethylene signaling after treatment with simulated herbivory and acquired enhanced resistance to larval feeding. As the biological significance of herbivory-induced accumulation of rice DPs remains unexplained, their physiological activities in *M. loreyi* were analyzed. The addition of momilactone B, a rice DP, to the artificial diet suppressed the growth of *M. loreyi* larvae. Altogether, this study revealed that BSR1 and herbivory-induced rice DPs are involved in the defense against chewing insects, in addition to pathogens.

## 1. Introduction

Plants can be damaged by both arthropod herbivores and microbial pathogens. Herbivores feed on crops by chewing or piercing-sucking, resulting in severe losses in agricultural production [1,2]. Furthermore, herbivores and pathogens can synergistically attack plants. Wounds formed by herbivory can promote opportunistic infections by microbial pathogens [3,4,5]. Many insects have been reported to act as vectors to help spread and infect pathogens [4,5]. Therefore, the development of simultaneous resistance to insect damage and disease is important.

Understanding the defense mechanisms of plants against biological stresses can provide solutions to developing crops that are resistant to pathogens and pests. In the interaction between plants and pathogenic microbes, plants sense pathogens via the recognition of microbial components and wound-derived molecules, termed microbe-associated molecular patterns (MAMPs) and damage-associated molecular patterns (DAMPs), respectively [6]. Many combinations of these patterns and corresponding plasma-membrane-localized pattern-recognition receptors have been reported [7]. For example, a family of endogenous peptides, termed plant elicitor peptides (Peps), function as DAMPs [8,9]. Peps are released from plants upon injury and recognized by Pep receptors (PEPRs), leucine-rich repeat (LRR) receptor-like kinases (RLKs), to activate defense responses [10]. Pep signaling is conserved in a wide range of plants, including dicotyledonous *Arabidopsis thaliana* (L.) Heynh and monocotyledonous *Oryza sativa* L. (rice) [8,11,12,13]. Following pattern recognition, plants mount a series of defense responses, including the production of reactive oxygen species (ROS), activation of mitogen-activated protein kinase (MAPK) cascades, upregulation of defense-related genes, and biosynthesis of secondary metabolites, resulting in increased resistance known as pattern-triggered immunity [14,15].

Similar to disease recognition, recent studies have revealed that plants recognize molecules derived from the feeding of insects [16]. As chewing herbivores feed, their oral secretions (OS) adhere to the plant. OS contain DAMPs and herbivore-associated molecular patterns (HAMPs), that is, metabolites and peptides produced during feeding and digestion [17,18,19]. *Vigna unguiculata* (L.) Walp. (cowpea) recognizes inceptin, a lepidopteran larvae-associated HAMP, via the membrane-localized LRR-receptor-like protein VuINR [20]. In the interaction between rice and the striped stem borer *Chilo suppressalis* Walker (Lepidoptera: Crambidae), a plasma-membrane-localized OsLRR-RLK1, whose ligand remains unknown, regulates defense responses elicited by *C. suppressalis*, such as activation of the MAPK cascade. Silencing OsLRR-RLK1 decreases rice resistance to *C. suppressalis* [21]. The soybean receptor-like kinases GmHAK1 and GmHAK2 mediate responses triggered by unidentified polysaccharides extracted from *Spodoptera litura* Fabricius (Lepidoptera: Noctuidae) OS [22].

In pattern-triggered immunity, protein kinases belonging to subfamily VII of the receptor-like cytoplasmic kinase (RLCK) family play crucial roles in intracellular phosphorylation signaling [23,24,25]. Following MAMP recognition, RLCKs are activated by various pattern-recognition receptor complexes and phosphorylate downstream factors such as the MAPK cascade to activate defense-related genes [26,27,28] and RBOH proteins to initiate an ROS burst [29,30,31]. *Broad-spectrum resistance 1* (*BSR1*) is a rice gene encoding an RLCK of subfamily VII [32,33]. In rice, defense responses triggered by MAMPs (chitin oligomers, peptidoglycan, and lipopolysaccharides) are mediated by BSR1, indicating that it is a key MAMP-signaling factor in rice [34,35]. Among many RLCKs, BSR1 is unique in that its overexpression mediates enhanced resistance. Overexpression of *BSR1* confers strong resistance against various fungal and bacterial pathogens in rice, sugarcane, *A. thaliana*, tomato, and torenia [32,36,37].

Similar to the contribution of RLCKs to disease response signaling, it was reported that Arabidopsis RLCK PBL27 mediates signal transduction downstream of AtHAK1, an RLK that recognizes the OS of *S. litura* larvae [22,38]. Although no RLCK has been reported to be involved in monocot plant–chewing herbivore interactions, we speculated that BSR1 might also regulate the defense responses elicited by chewing herbivores. If so, *BSR1* overexpression may confer resistance against both herbivores and pathogenic microbes. Here, we investigated the role of BSR1 in the molecular interactions between rice and the Gramineae chewing herbivore pest *Mythimna loreyi*.

## 2. Results

### 2.1. BSR1 Contributes to Defense Responses Triggered by Larval OS

**Figure 1:**
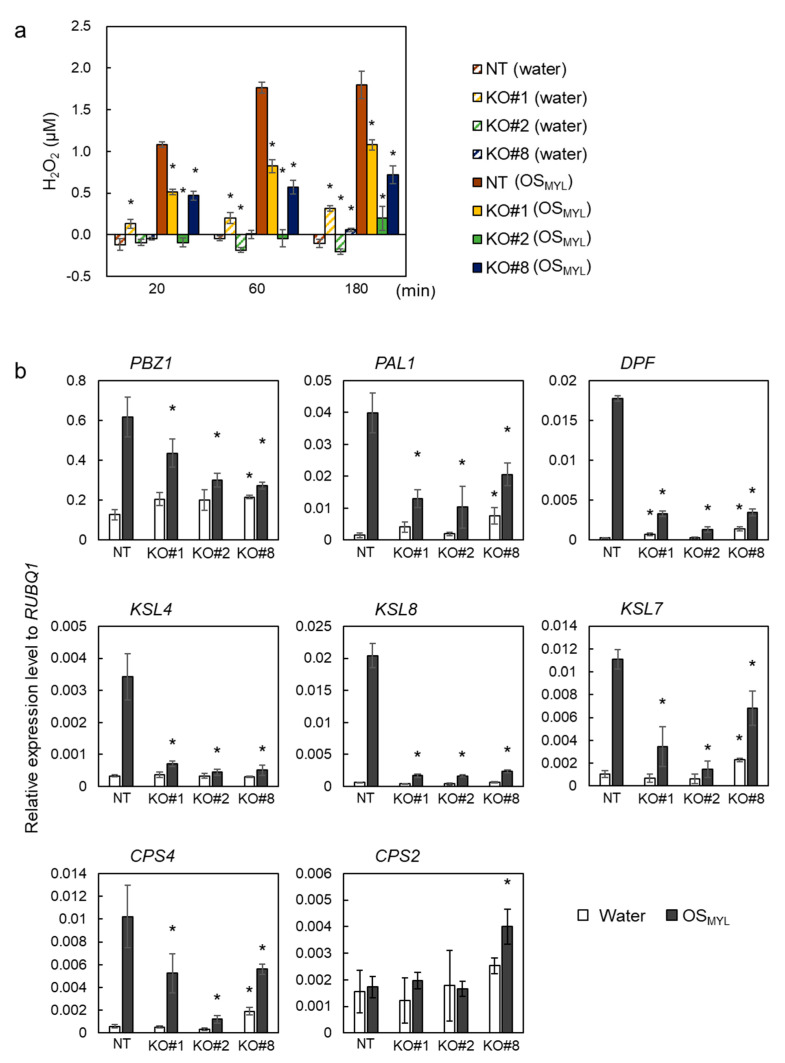
Knockouts of *BSR1*-impaired defense responses in rice cell cultures treated with *Mythimna loreyi* oral secretions. Suspension-cultured cells were treated with oral secretions (OS) (500-fold dilution). (**a**) Time course of H_2_O_2_ production in the cell culture. A 2 µL volume of OS was added to 1 mL medium containing cells. The H_2_O_2_ concentrations were measured before (0 min) and 20, 60, and 180 min after treatment and calculated by subtracting the value measured at 0 min from that at each time point. (**b**) Transcriptional activation of defense-related genes in suspension-cultured rice cells. The *PBZ1*, *PAL1*, *DPF*, *KSL4*, *KSL8*, *KSL7*, *CPS2*, and *CPS4* transcript levels 3 h after treatment with OS were normalized against *RUBQ1* internal control levels. Asterisks indicate significant differences between the values of NT and those of the other lines (Dunnett’s test; * *p* < 0.05). Values are presented as the mean ± standard deviation of three biological replicates in one representative experiment. The experiments were conducted twice using independently induced suspension-cultured cells, and similar results were obtained (Figure S1). Water, treated with sterile water; OS_MYL_, treated with *Mythimna loreyi* OS; KO, *BSR1-*knockout line; NT, non-transgenic line (Nipponbare).

To assess whether BSR1 functions in defense responses against chewing in lepidopteran larvae, we evaluated OS-induced defense responses in *BSR1*-knockout (KO) lines. OS were collected from *M. loreyi* larvae fed on rice leaves (referred to hereafter as OS_MYL_). Three independent lines containing homozygous frameshift mutations in *BSR1* [34] were used as *BSR1*-KO lines. Suspension-cultured cells were derived from KO lines and a non-transgenic (Nipponbare) line and treated with OS_MYL_. After treatment, H_2_O_2_ concentrations in the liquid-cultivation media of each cell line were determined (Figure 1a). During the measurements taken after treatment with OS_MYL_, H_2_O_2_ levels in all three *BSR1*-KO lines were significantly lower than those in non-transgenic lines (Figure 1a and Figure S1a). KO cells accumulated 47% or lower amounts of H_2_O_2_ compared with non-transgenic cells 60 min after treatment. At 180 min after treatment with OS_MYL_, the transcript levels of some key defense-related genes were determined. These included the representative defense marker gene, *probenazole inducible protein 1* (*PBZ1*); the flavonoid phytoalexin and lignin biosynthetic gene, *phenylalanine ammonia-lyase 1* (*PAL1*); the key transcription factor gene for diterpenoid phytoalexin (DP) biosynthesis, *diterpenoid phytoalexin factor* (*DPF*); and the DP biosynthetic genes, *kaurene synthase-like 4* (*KSL4*), *KSL7*, *KSL8*, *copalyl diphosphate synthase 2* (*CPS2*), and *CPS4* (Figure 1b, Figure S1b and S2 ). Knocking out *BSR1* decreased the transcript levels of these defense-related genes in response to OS_MYL_ treatment. The expression of *CPS2* appeared constitutive and was not affected by *BSR1* knockout. These results show that BSR1 contributes to the activation of rice defense responses induced by OS_MYL_.

**Figure 2:**
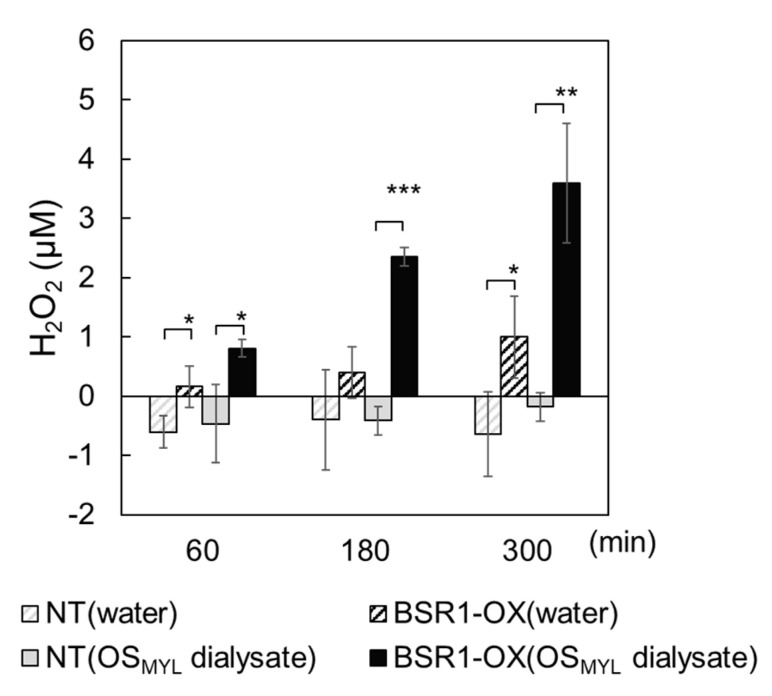
Overexpression of *BSR1* enhanced oral-secretion-induced reactive oxygen species bursts in rice leaf strips. Leaf strips were treated with water or dialyzed OS_MYL_. The H_2_O_2_ concentrations were measured before (0 min) and 60, 180, and 300 min after treatment and calculated by subtracting the value measured at 0 min from that at each time point. Asterisks indicate significant differences between the values of NT and those of the other lines (*t*-test; * *p* < 0.05, ** *p* < 0.01, and *** *p* < 0.001). Values are presented as the mean ± standard deviation of three biological replicates in one representative experiment. The experiments were conducted twice, and similar results were obtained (Figure S4). Water, treated with sterile water; OS_MYL_ dialysate, treated with dialyzed oral secretions; BSR1-OX, *BSR1*-overexpressing line; NT, non-transgenic line (Nipponbare).

Furthermore, the OS_MYL_-triggered responses in *BSR1*-overexpressing (OX) leaf strips were analyzed. BSR1-OX-5, a previously generated *BSR1*-OX line [32], was used. Consistent with a previous report [39], OS_MYL_ inhibited the luminol-dependent detection of H_2_O_2_, resulting in no detectable increase in H_2_O_2_ levels in the OS-treated leaf strips. The addition of OS_MYL_ decreased the level of background chemiluminescence in the absence and presence of H_2_O_2_ (Figure S3). This suggests that OS_MYL_ possesses chemiluminescence-quenching activity, rather than ROS-scavenging activity. A dialyzed fraction of OS_MYL_ was then used to avoid inhibitory activity, according to a previous report [39]. At 60, 180, and 300 min after treatment with the dialysate of OS_MYL_, *BSR1-*OX leaf strips accumulated a significantly greater amount of H_2_O_2_ compared with non-transgenic leaf strips (Figure 2).

### 2.2. BSR1 Mediates the OsPep-Triggered Defense Response in Rice

**Figure 3:**
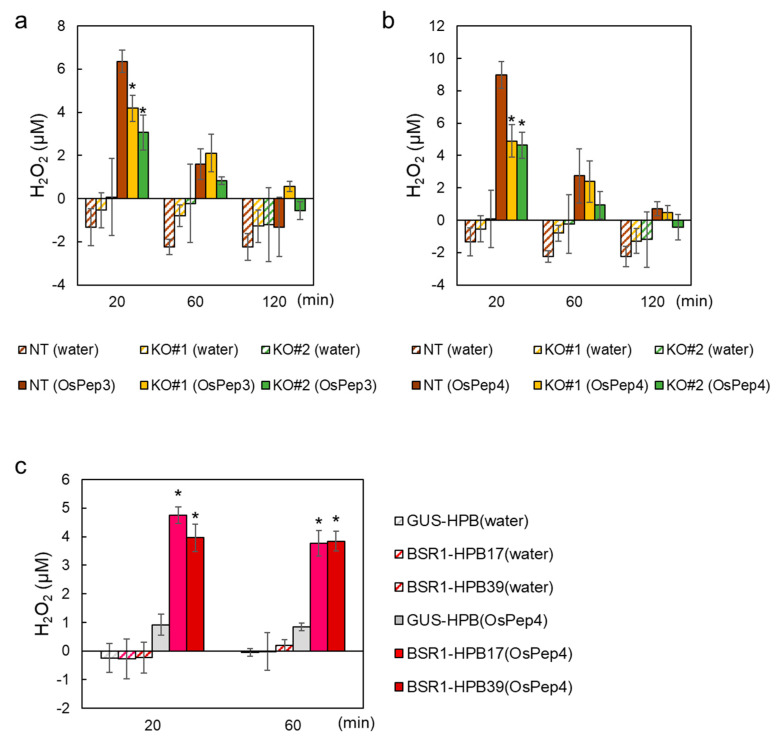
BSR1 regulates OsPep3- and OsPep4-triggered defense response in cultured cells. (**a**,**b**) *BSR1*-KO suspension-cultured cells were treated with OsPep3 (**a**) and OsPep4 (**b**). The H_2_O_2_ concentrations were measured before (0 min) and 20, 60, and 120 min after treatment and calculated by subtracting the value measured at 0 min from that at each time point. (**c**) *BSR1*-overexpressing suspension-cultured cells were treated with OsPep4, and H_2_O_2_ concentrations were measured before as well as 20 and 60 min after treatment. Asterisks indicate significant differences between values of NT and KO lines or between those of the GUS-HPB control and BSR1-HPB lines under the same treatment conditions (Dunnett’s test; * *p* < 0.05). Values are presented as the mean ± standard deviation of three biological replicates in one representative experiment. The experiments were conducted twice using independently induced suspension-cultured cells, and similar results were obtained (Figure S5). Water, treated with sterile water; KO, *BSR1-*knockout line; NT, non-transgenic line (Nipponbare).

To understand the mechanism underlying BSR1-mediated responses to OS, a component of OS that is perceived upstream of BSR1 was explored. OS_MYL_-induced defense responses are augmented in the presence of rice-endogenous DAMPs known as OsPeps. *OsPROPEP3* and *OsPROPEP4*, precursors of OsPep3 and OsPep4, respectively, were induced in rice leaves by mechanical wounding and by simulated herbivory [12]. We then tested whether BSR1 contributes to the responsivity of the rice peptide DAMPs OsPep3 and OsPep4. Suspension-cultured cells were treated with rice Peps, and Pep-triggered H_2_O_2_ production was quantified. H_2_O_2_ production was moderately but significantly suppressed by knockout of *BSR1* (Figure 3a,b and Figure S5a,b). The contribution of BSR1 to rice responses to OsPeps was further validated using suspension-cultured cells overexpressing tagged BSR1 (*BSR1-HPB*) or a tagged control protein (*GUS-HPB*). At 20 and 60 min after treatment with OsPep4, *BSR1-HPB*-overexpressing cells produced greater amounts of H_2_O_2_ than control cells (Figure 3c and Figure S5c). These results indicate that BSR1 contributes to OsPep-triggered defense responses in rice and that overexpression hyperactivates Pep signaling.

### 2.3. Overexpression of BSR1 Enhances Phytoalexin Production

Since BSR1 positively regulated OS_MYL_- and DAMP-triggered responses in cultured rice cells and leaf strips (Figure 1, Figure 2 and Figure 3), we presumed that *BSR1*-OX plants could show higher defense responses against herbivory. However, the above-described *BSR1*-OX lines in which *BSR1* was expressed under the strong maize ubiquitin promoter displayed a decreased germination rate [32], and the timing of germination was unaligned. Hence, it was difficult to match growth with the control line. Therefore, we used *BSR1*-OX lines #22 and #42, in which *BSR1* was expressed under the moderate rice *Ubi7* promoter and of which the germination was normal.

**Figure 4:**
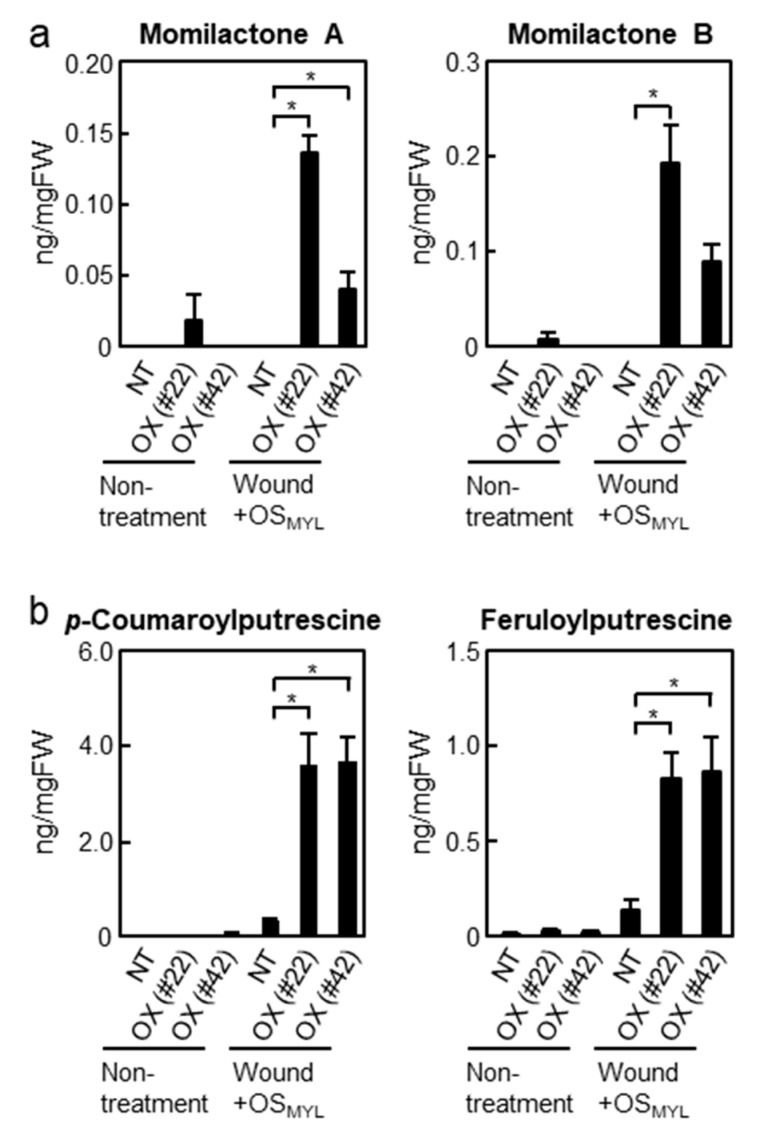
Accumulation of defense-related metabolites was promoted by *BSR1* overexpression. Amounts of momilactones (**a**) and phenolamides (**b**). Rice leaves were collected 24 h after treatment for the metabolite assay. Data are shown as mean ± SE (independent biological replicates, *n* = 4). Statistical differences were analyzed using Dunnett’s test (* *p* < 0.05). NT, non-transgenic line (Nipponbare); OX (#22) and OX (#42), *BSR1*-overexpressing lines #22 and #42.

To assess the defense responses induced by simulated herbivory, fully developed rice leaves attached to intact rice were first wounded with a serrated tracing wheel, whereafter the wounds were treated with diluted OS_MYL_ (hereafter referred to as simulated herbivory). The secondary metabolites DPs (momilactone A and B) and phenolamides (*p*-coumaroylputrescine (CoP) and feruloylputrescine (FP)) were analyzed 24 h after simulated herbivory treatment. Momilactone A and B levels were only significantly increased after simulated herbivory in *BSR1*-OX plants, whereas low accumulation was observed in the non-transgenic plants upon simulated herbivory (Figure 4a). Additionally, phenolamides were highly accumulated in *BSR1-*OX plants upon simulated herbivory compared with those in non-transgenic plants (Figure 4b). These results suggest that the overexpression of *BSR1* enhances phytoalexin production.

### 2.4. Overexpression of BSR1 Alters the Level of Volatile Organic Compounds

**Figure 5:**
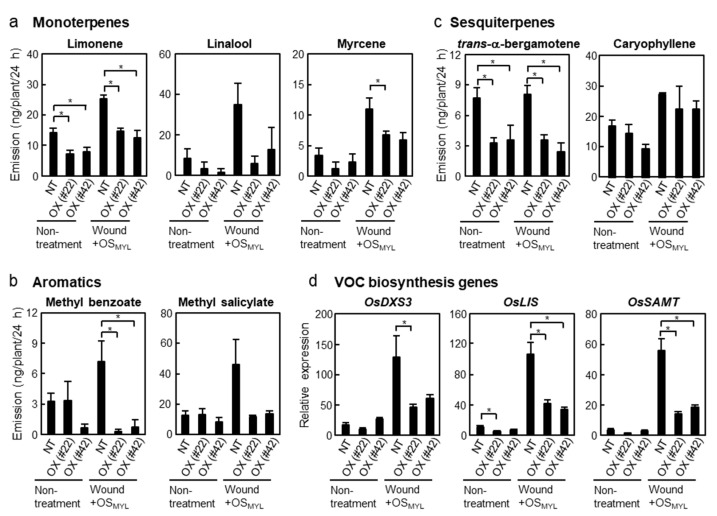
*BSR1*-overexpression reduced headspace volatile organic compounds emitted from rice. (**a**–**c**) Volatile organic compounds (VOCs) were collected for 24 h after treatment and measured via GC-MS (independent biological replicates, *n* = 4). (**d**) Transcript levels of VOC biosynthesis genes were measured at 1 h after treatment in rice leaves (independent biological replicates, *n* = 6). Data are shown as mean ± SE. Statistical differences were analyzed using Dunnett’s test (* *p* < 0.05). NT, non-transgenic line (Nipponbare); OX (#22) and OX (#42), *BSR1*-overexpressing lines #22 and #42.

Plants emit numerous volatile organic compounds (VOCs) during herbivory. Herbivore-induced plant volatiles serve to attract natural enemies to herbivores, a phenomenon called indirect defense [16]. We measured VOCs released from rice leaves into the headspace for 24 h. Monoterpenes (limonene, linalool, and myrcene) accumulated after simulated herbivory, although their amounts were clearly or marginally decreased in *BSR1*-OX plants (Figure 5a). Similarly, the levels of aromatic compounds (methyl benzoate and methyl salicylate) were suppressed in *BSR1*-OX plants upon simulated herbivory (Figure 5b). Among sesquiterpenes, *trans*-α-bergamotene showed lower accumulation in *BSR1*-OX than in the non-transgenic line, whereas caryophyllene showed no significant difference by *BSR1* overexpression upon simulated herbivory (Figure 5c). In addition, we measured the transcript levels of biosynthesis genes for some VOCs at 1 h after simulated herbivory. As with the VOC levels, linalool biosynthesis genes (*OsLIS* and *OsDXS3*) were decreased in *BSR1*-OX plants compared with the non-transgenic plants (Figure 5d). Likewise, the transcript levels of *OsSAMT*, which is involved in methyl salicylate biosynthesis, were lower in *BSR1-*OX than in the non-transgenic plants (Figure 5d). Overall, the expression profiles of these biosynthetic genes resembled the emission profiles of the corresponding metabolites. Altogether, multiple accumulation patterns of rice VOCs showed a tendency to be suppressed by *BSR1* overexpression.

### 2.5. Defense-Related Gene Expression and Mechanical Defenses

Proteinase inhibitors (PIs) are typical anti-herbivore proteins, and we measured the transcript levels of wound-induced *PI* genes [40]. *WIPI* and *PI (Os1g0127600)* transcripts were reduced by *BSR1* overexpression (Figure S6). Another defense-related gene, *OsPR1b*, was highly expressed in *BSR1-*OX (Figure S6). Together with the transcriptional analysis of biosynthesis genes, *BSR1* overexpression largely modulated the expression of defense-related genes regulated by herbivory.

We further examined whether *BSR1* overexpression affects mechanical defense in rice plants. Silicified trichomes have been reported to reduce the performance of chewing herbivores such as *M. loreyi* [41]. Scanning electron microscopy analysis of rice leaves revealed no significant difference in the number of trichomes between non-transgenic and *BSR1*-OX plants (Figure S7). In addition to these observations, histochemical staining of lignin in leaf cross-sections showed no obvious differences between the non-transgenic and *BSR1*-OX plants (Figure S8). Overexpression of *BSR1* is, therefore, unlikely to affect the strength of mechanical defenses.

### 2.6. Phytohormone Biosynthesis in BSR1-OX

**Figure 6:**
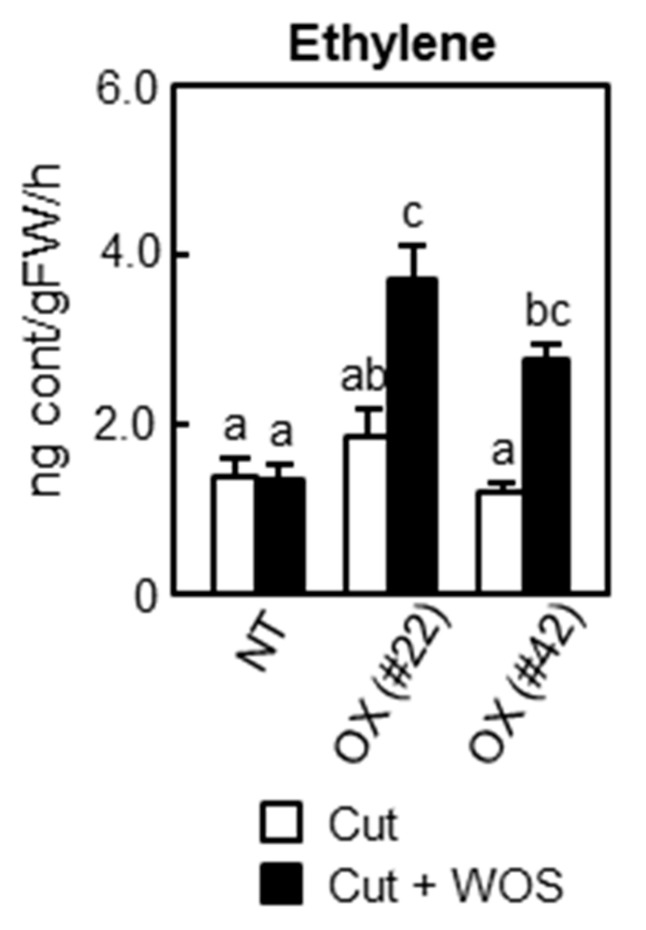
Ethylene production in *BSR1*-OX. The youngest fully developed leaves cut out from six-week-old plants were treated with WOS (Cut + WOS) or non-treated (Cut). Subsequently, ethylene levels were determined via GC-FID in the headspace of cut leaves incubated for 24 h in closed glass containers. Different letters indicate significant differences (*p* < 0.05) calculated using one-way ANOVA followed by Tukey’s HSD test. Data are shown as mean ± SE (independent biological replicates, *n* = 6). WOS, wound + OS_MYL_; NT, non-transgenic line (Nipponbare); OX (#22) and OX (#42), *BSR1*-overexpressing lines #22 and #42.

To explore regulatory mechanisms underlying herbivory-triggered defense responses in *BSR1*-OX plants, we measured their hormonal changes. Jasmonates are typical regulatory hormones for defenses triggered by herbivory and wounding. Although the production of jasmonates was promoted by simulated herbivory in non-transgenic and *BSR1*-OX plants, their accumulation levels were similar (Figure S9). Abscisic acid levels showed no apparent differences in accumulation between non-transgenic and *BSR1-*OX plants. Although salicylic acid levels significantly varied in some cases, they did not show consistent changes in two independent *BSR1-*OX plants compared with that in the non-transgenic plant. Finally, we measured the amount of ethylene because of the similar phenotype of *BSR1*-OX and ethylene-exposed rice plants, such as suppression of VOCs (Figure 5a–c) and reduced expression of VOC biosynthetic genes (Figure 5d) [42]. When we compared the amount of ethylene between non-transgenic and *BSR1*-OX plants, the *BSR1*-OX plants produced more ethylene at 24 h after simulated herbivory treatment (Figure 6). These results suggest that ethylene could function as a regulator to modulate defense responses downstream of BSR1.

### 2.7. BSR1 Overexpression and Momilactone B Suppress the Larval Performance of M. loreyi

**Figure 7:**
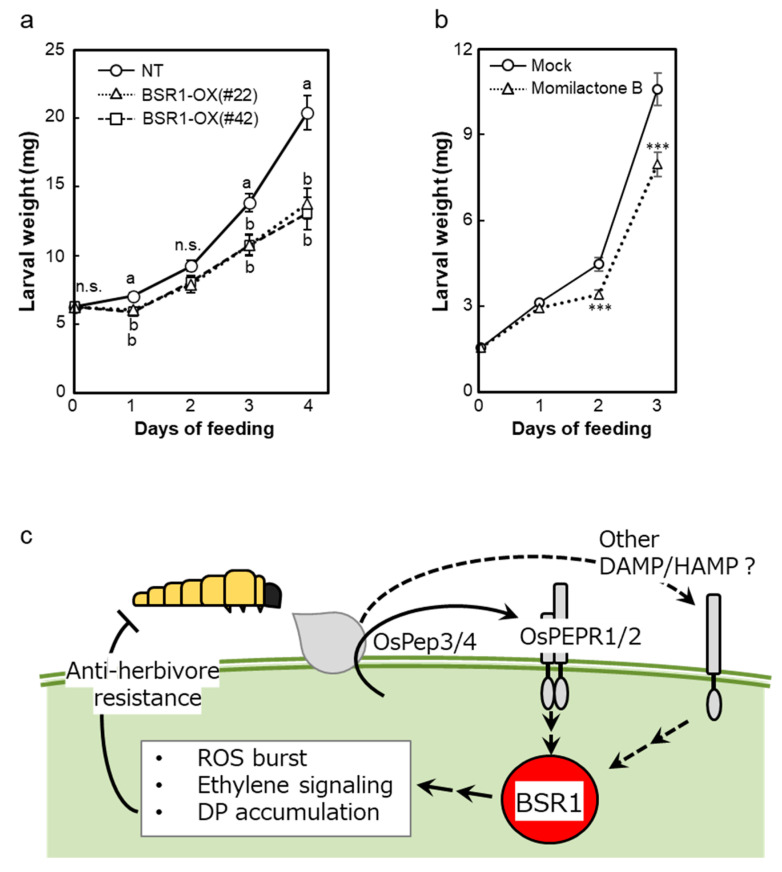
Larval growth performance of *Mythimna loreyi*. (**a**) *BSR1* overexpression in rice decreases the performance of *M. loreyi* larvae. Larval masses are shown as mean ± SE (independent biological replicates, *n* = 19–25). Different letters indicate significant differences (*p* < 0.05) calculated using ANOVA followed by Tukey’s HSD test in each time point. n.s., not significant. (**b**) Biological activity of momilactone B against *M. loreyi*. The *M. loreyi* larvae were individually fed on mock or momilactone B-containing artificial diets. Data are shown as mean ± SE (independent biological replicates, *n* = 33–35). Statistical differences were analyzed in each time point using Student’s *t*-test (*** *p* < 0.001). (**c**) Schematic model of insect resistance brought about by *BSR1* overexpression. BSR1 mediates defense signaling in response to OsPep (and other oral secretion components) and positively regulates downstream responses, including the biosynthesis of diterpenoid phytoalexins (DPs). The resulting accumulation of DPs suppresses larval growth. NT, non-transgenic line (Nipponbare); BSR1-OX (#22) and (#42), *BSR1*-overexpressing lines #22 and #42.

BSR1 mediates defense responses triggered by OS_MYL_ and DAMPs in rice cells (Figure 1, Figure 2 and Figure 3). However, overexpression of *BSR1* altered multiple defense responses upward or downward in rice leaves (Figure 4, Figure 5 and Figure S6). To test the overall effect of *BSR1* overexpression on insect performance, feeding experiments were implemented. The larvae of *M. loreyi* in clip cages were placed on leaf blades of non-transgenic or *BSR1*-OX rice plants. Overexpression of *BSR1* significantly suppressed the increase in weight of *M. loreyi* larvae (Figure 7a and Figure S10a). Because defense-related metabolites were significantly induced in *BSR1*-OX plants compared with those in non-transgenic plants (Figure 4a,b), we initially assumed that these metabolites could be a causal component for the suppression of larval performance. However, our previous work indicated that feruloylputrescine had no apparent effect on the chewing insect larvae of *S. mauritia* Boisduval (Lepidoptera: Noctuidae) and *Parnara guttata* Bremer & Grey (Lepidoptera: Hesperiidae) [43]. Moreover, the effects of momilactones on herbivores have not been demonstrated. Momilactone induction has been observed during herbivory by sucking insects, but herbivory by chewing insects resulted in weak induction of momilactones in non-transgenic rice plants (Figure 5a) [39,43,44]. Therefore, we hypothesized that the reduction in larval mass could be due to hyperaccumulation of momilactones. To address this possibility, an artificial diet was mixed with momilactone B purified from rice hulls and used for the larval performance assay with *M. loreyi*. The assay revealed that momilactone B inhibited *M. loreyi* growth two or three days after feeding (Figure 7b and Figure S10b). Accordingly, hyperaccumulation of momilactones upon herbivory most likely suppresses *M. loreyi* larvae feeding on *BSR1*-OX rice plants.

## 3. Discussion

In this study, *BSR1* knockout suppressed the ROS burst and transcriptional activation of defense-related genes triggered by OS_MYL_ (Figure 1). These results suggest that BSR1 mediates the activation of defense responses against chewing herbivores in monocot rice. Furthermore, the knockout also decreased the responses triggered by OsPep3 and OsPep4 (Figure 3a,b). Pep peptides are recognized by the membrane-localized receptors PEPR1 and PEPR2 to activate a cytoplasmic signaling pathway [10]. The signaling pathway is reported to comprise RLCKs in Arabidopsis [45,46], although no monocot RLCK has been reported to contribute to Pep-triggered responses. Our experiments revealed that BSR1, an RLCK, mediates the Pep-triggered responses in monocot rice plants. Based on our results, the molecules recognized upstream of BSR1 in the OS response may be OsPep peptides or, alternatively, the HAMPs pathway may have crosstalk with the OsPep pathway during downstream signal transduction via BSR1 (Figure 7c). In the interaction between Arabidopsis and larvae of the chewing herbivore *S. littoralis*, Pep-insensitive mutant plants exhibited reduced resistance and a lowered level of jasmonic acid (JA) production triggered by larval OS [47]. Rice leaves constitutively express three *OsPROPEPs*, OsPep precursors; furthermore, *OsPROPEP3* is induced during herbivory [12]. The OS_MYL_ used in our study may contain plant-derived OsPep peptides because it was obtained from *M. loreyi* larvae fed with rice leaves. However, it has not been proven that OS_MYL_ contains OsPep peptides, and various elicitors are present in the OS of chewing herbivores [19]. Therefore, further investigation is required to identify the OS_MYL_ component(s) upstream of BSR1.

This study further revealed that *BSR1*-OX rice plants displayed enhanced resistance to feeding by *M. loreyi* larvae (Figure 7a and Figure S10a). Overexpression of *BSR1* augmented the defense responses to treatment with OsPep4 (Figure 3c), consistent with the previously reported enhancement of MAMP-triggered responses in *BSR1*-OX cells [35]. There are also previous findings on a correlation between Pep signaling and anti-herbivore resistance [10]. In Arabidopsis and maize, Pep peptides elicit anti-herbivore responses [11,47]. In the interaction between rice and *M. loreyi*, OsPep peptides amplify OS-triggered defense responses, including the ROS burst and biosynthesis of secondary metabolites [12]. Shen et al. [13] reported that exogenous application of OsPep3 improved rice resistance to *Nilaparvata lugens* Stål (Homoptera: Delphacidae) (brown planthopper), a sucking insect. Together with these findings, the enhancement of anti-herbivore resistance in *BSR1*-OX plants could be achieved by hyperactivation of Pep-triggered defense signaling (Figure 7c).

Our study also revealed that *BSR1*-OX rice plants produced higher levels of the gas phytohormone ethylene than those in non-transgenic plants after wounding and treatment with OS_MYL_ (Figure 6). Analysis of VOCs provided further evidence for the activation of ethylene signaling. In a previous report, exposure to ethylene resulted in the suppression of VOCs, such as linalool, limonene, and methyl salicylate, in rice leaves upon simulated herbivory [42]. Similar to VOCs, transcripts of the VOC biosynthetic genes, *OsLIS*, *OsDXS3*, and *OsSAMT*, were suppressed by direct application of ethylene [42]. Consistent with the greater levels of ethylene (Figure 6), we found a suppressed emission of VOCs triggered by simulated herbivory in *BSR1*-OX plants, which also had a lower level of *OsLIS*, *OsDXS3*, and *OsSAMT* transcripts in response to OS_MYL_ (Figure 5). These data indicate that ethylene signaling is likely to be superactivated by the overexpression of *BSR1*.

In principle, VOC biosynthesis in rice is positively regulated by jasmonates [48]. As JA and JA-Ile levels appeared normal in the *BSR1*-OX plants upon simulated herbivory (Figure S9), *BSR1*-OX-induced ethylene is likely to act as a negative regulator downstream of jasmonate signaling, thus effectively reducing VOC levels (Figure 5). Further, ethylene may directly enhance some other defense pathways, such as momilactone accumulation and/or ROS levels, affecting herbivore performance. Ethylene emissions occur upon insect infestation in a variety of plants, such as *Nicotiana attenuata* Torr. ex S. Watson, *A. thaliana*, *Glycine max* (L.) Merr. (soybean), *Zea mays* L. (maize), and rice [38,49,50], and ethylene emission triggered by wounding and OS varies with the growth stage of the plant [42,51]. The effects of ethylene on herbivore resistance have been extensively reported, although there are inconsistent findings on the correlation between ethylene signaling and herbivore resistance. Arabidopsis mutants defective in ethylene signaling are more resistant to *S. exigua* Hübner (Lepidoptera: Noctuidae) and *S. littoralis* Boisduval (Lepidoptera: Noctuidae) larvae, suggesting that ethylene signaling negatively affects resistance to chewing herbivores [52,53]. In contrast, ethylene signaling mediates the defense responses induced by unidentified elicitor(s) extracted from the larval OS of *S. litura* [22]. In monocots, maize treated with an inhibitor of ethylene biosynthesis or signaling exhibited greater susceptibility to *S. frugiperda* Smith (Lepidoptera: Noctuidae) [54]. In rice, the ethylene biosynthetic pathway positively contributes to resistance against the striped stem borer (*C. suppressalis*) [55]. Our data support that ethylene has a positive effect on resistance to chewing herbivores in rice.

We further investigated the barriers against herbivory in *BSR1*-OX plants. Overexpression of *BSR1* markedly increased the accumulation of secondary metabolites in leaf blades treated with simulated herbivory, although it did not potentiate the emission of VOCs, expression of protease inhibitors, formation of trichomes, or accumulation of lignin (Figure 4, Figure 5 and Figures S6–S8). After treatment with simulated herbivory, *BSR1*-OX rice leaves accumulated higher levels of CoP and FP, two major phenolamides produced in rice [56]. This implies that *BSR1*-OX plants respond more strongly to herbivory. However, these phenolamides would not contribute to the BSR1-mediated enhancement of resistance; FP possesses no detectable insecticidal activity against chewing insects [43] and CoP showed no significant activity in our preliminary experiments.

Further quantification of the rice DPs momilactone A and momilactone B in *BSR1*-OX plants (Figure 4a) shed light on their contribution to anti-herbivore resistance. Plants produce phytoalexins, including DPs, under various stressful conditions. Numerous studies have reported that rice DPs contribute to resistance to microorganisms, such as the fungal pathogen *Pyricularia oryzae* and the bacterial pathogen *Xanthomonas oryzae* pv. *oryzae* [15,57,58]. Rice DPs also contribute to resistance against root-knot nematodes [59]. However, the relationship between rice DPs and herbivory resistance is unclear. Feeding by the sucking insect white-backed planthopper *Sogatella furcifera* Horváth (Hemiptera: Delphacidae) induces the production of momilactone A [44]. Alamgir et al. [43] previously reported that rice accumulates momilactone A and momilactone B upon sucking by the brown planthopper but does not accumulate these DPs upon feeding by the larvae of two chewing herbivores, the lawn armyworm (*S. mauritia*) and rice skipper (*P. guttata*). In the experiment using *M. loreyi*, leaf discs of Nipponbare rice accumulated momilactone A in response to OS_MYL_ [12]. Because insect herbivores can act as vectors for microbial pathogens [3,4,5], herbivory-induced DPs have been described as a factor conferring resistance to subsequent infection by pathogens and a direct effect on herbivores has not been verified [15,44]. In this study, overexpression of *BSR1* promoted the accumulation of momilactone A and momilactone B under simulated herbivory conditions, and *BSR1*-OX plants displayed improved resistance to *M. loreyi* (Figure 4a and Figure 7a). Contrary to the previous report using OS_MYL_-treated leaf discs [12], non-transgenic plants did not accumulate momilactones against simulated herbivory with OS_MYL_ (Figure 4a). This discrepancy could be because the simulated herbivory we employed was too weak to induce the production of DPs to detectable levels in plants. In cultured cells, OS_MYL_ clearly induced the expression of the biosynthetic genes *CPS4* and *KSL4*, which are responsible for the biosynthesis of momilactones, and *DPF*, the key transcription factor for DP biosynthesis [60] (Figure 1b). The expression levels of these genes were reduced in *BSR1*-KO mutants (Figure 1b). Altogether, these results suggest that BSR1 mediates herbivory-triggered biosynthesis of momilactones (and other DPs) in rice. Therefore, we further investigated the biological significance of rice DPs. The addition of momilactone B to artificial diets resulted in poor larval growth (Figure 7b and Figure S10b), clearly showing that it has direct toxicity to the larvae of *M. loreyi* rather than resistance-inducing activity. Although we demonstrated only acute toxicity of momilactone B in an experimental environment, it may possibly retard larval growth and reproduction in the long-term interaction between rice and *M. loreyi* in the field. As no findings have been reported on the biological implications of rice DPs for interactions between the plant and chewing herbivores, the detailed physiological mechanism is unknown. In mammals, there is a report that momilactone B is cytotoxic to human colon cancer cells [61]. Cytotoxicity is mediated by the induction of cell cycle arrest at the G1 phase and apoptotic cell death [62]. The direct inhibitory effect on growth of *M. loreyi* (Figure 7b and Figure S10b) may be achieved by a similar mechanism. Taken together, our data suggest that momilactone B (and other DPs) can serve as a form of direct defense against chewing herbivores, especially in plants that express high levels of BSR1.

Rice does not necessarily accumulate momilactones upon feeding by chewing herbivores [43]. Consistent with this report, rice (Nipponbare) did not accumulate detectable levels of momilactones after treatment with simulated herbivory using *M. loreyi* OS (Figure 4a). Meanwhile, it has been reported that herbivorous insects and pathogenic microbes avoid triggering host plant defense responses in various ways [19]. For example, Vu-In^-A^, a peptidic effector contained in the OS of the chewing herbivore *Anticarsia gemmatalis* Hübner (Lepidoptera: Noctuidae) larva, suppresses HAMP-triggered defense responses, including ethylene emission [63]. Similarly, in the interaction between rice and the brown planthopper, the salivary effector NlSEF1 suppresses H_2_O_2_ production and promotes feeding [64]. In our experiments, *BSR1*-OX plants displayed overactivated responses triggered by OS and DAMPs (Figure 2 and Figure 3c). Therefore, the following hypothesis seems plausible: *M. loreyi* (and other chewing herbivores) are also adapted to avoid activating the host defense in non-transgenic (wild-type) rice, whereas excessively induced responses, such as accumulation of DPs, in OS-hyperresponsive *BSR1*-OX rice enhances resistance against chewing herbivores (Figure 7c). To conclude, our study showed that an RLCK BSR1 and its downstream secondary metabolites, rice DPs, are involved in the resistance against multiple biotic stressors, including chewing insects, in addition to pathogens. There are only a few examples in which a single gene confers enhanced resistance to both herbivorous arthropods and pathogenic microbes. To the best of our knowledge, no RLCK-encoding genes other than *BSR1* have been reported to confer such resistance. However, because many RLCK-encoding genes are distributed in a wide variety of plants [23,24], unidentified RLCKs may be conserved as functional orthologues of BSR1. Although the model shown in Figure 7c and its applicability range should be further verified experimentally, this study sheds light on the plant resistance mechanisms employed against a wide range of biotic stressors.

## 4. Materials and Methods

### 4.1. Plant and Lepidopteran Materials

The *O. sativa* L. cv. Nipponbare (rice) was used as the wild-type (non-transgenic) plant material. The independent lines *bsr1-1*#13-1 (KO#1), *bsr1-2*#16-2 (KO#2), and *bsr1-8*#5-1 (KO#8), generated from Nipponbare using the CRISPR/Cas9 system in our previous study [34], were used as *BSR1*-KO lines. BSR1-OX-5 is a previously generated *BSR1*-OX line [32]. BSR1-HPB:OX17 (BSR1-HPB17) and BSR1-HPB:OX39 (BSR1-HPB39) lines [35] were used to prepare *BSR1*-overexpressing suspension-cultured cells. GUS-HPB:OX6 line (GUS-HPB) [35] was used as the control protein-overexpressing line. The HPB tag is a tandem protein tag composed of a hemagglutinin epitope, PreScission protease cleavage site, and biotin carboxyl carrier protein domain [65].

Germination in *BSR1*-OX lines was poor [32]. Therefore, some experiments used *BSR1*-OX lines #22 and #42 that were generated by using the moderate rice *Ubi7* promoter rather than the strong maize ubiquitin promoter. In detail, *WRKY45* cDNA of the *P_OsUbi7_*:*WRKY45*:*TT* plasmid [66,67] was replaced with *BSR1* cDNA, and the transgenic rice lines were generated from Nipponbare with the resulting plasmid using an *Agrobacterium*-mediated procedure [68]. Rice seeds of the Nipponbare and *BSR1*-OX lines #22 and #42 were placed in germination trays with Kumiai Ube Baido No. 2 nutrient-rich soil (MC Ferticom, Tokyo, Japan). After 2–3 weeks, the seedlings were transferred to larger pots with paddy field soil mixed with Kumiai Ube Baido No. 2 soil (20–30%).

A generalist herbivore, *M. loreyi*, was collected in Kurashiki (Okayama Prefecture, Japan) and reared under laboratory conditions, as described previously [39].

### 4.2. Collection of Oral Secretions

Oral secretions (OS_MYL_) were collected from 4–6th instar *M. loreyi* larvae fed on rice leaves for at least three days before OS collection [39]. The OS were collected with polypropylene tubing maintained under a mild vacuum and accumulated in a collection tube inserted in the vacuum path. Larvae were held between fingertips and mechanically disturbed with a tip of the polypropylene tube to induce regurgitation. The high-molecular-weight fraction of OS_MYL_ was prepared using dialysis tubing (3500 MW cut-off; BioDesign Inc., Carmel, NY, USA) as previously described [39].

### 4.3. Preparation of Suspension-Cultured Cells and Leaf Strips

The induction of suspension-cultured cells was performed according to a previously described method [35,69]. Briefly, the cells were prepared as follows. Rice calli were liquid-cultivated at 28 °C with shaking at 120 rpm in modified liquid N6 medium [30 g/L sucrose; 4.1 mg/L N6 salt (Wako Pure Chemical Corporation, Osaka, Japan); 2 mg/L glycine; 0.5 mg/L nicotinic acid; 0.5 mg/L pyridoxine HCl; 1 mg/L thiamine HCl; 100 mg/L myo-inositol; 1 mg/L 2,4-dichlorophenoxyacetic acid; 23.4 mg/L MnSO_4_·4H_2_O; and pH 5.8] [35] for a day. The liquid-cultivation samples were filtered through a mesh to suspend cells. After cultivation for three days, 100 mg of the resulting suspension-cultured cells and 1 mL fresh modified liquid N6 medium were dispensed into a 2 mL microtube whose lid had two pinholes. After incubation at 28 °C with shaking at 800 rpm overnight, the suspension-cultured cells were used for experiments. Leaf strips were made by cutting young seventh leaf blades using bundled razor blades, as previously described [35,70]. The leaf strips were incubated in sterile water at 28 °C with shaking at 90 rpm and used for experiments.

### 4.4. Measurement of H_2_O_2_

Suspension-cultured cells (100 mg) in modified liquid N6 medium and two leaf strips in sterile water were treated with the following elicitors at the indicated final concentrations: 500-fold diluted OS_MYL_, 100-fold diluted dialysate of OS_MYL_, 100 nM OsPep3, 100 nM OsPep4, or sterile Milli-Q water (as mock treatment). OsPep3 and OsPep4 were purchased from Pepmic (Suzhou, China) following custom synthesis. H_2_O_2_ concentration was measured at the indicated times using a previously described luminol-dependent chemiluminescence assay using luminol (Nacalai Tesque, Kyoto, Japan) and a TD-20/20 luminometer (Turner Designs, San Jose, CA, USA) [35,69,71]. A standard curve was constructed to calculate the concentration of H_2_O_2_. To assess the inhibitory activity of OS_MYL_ against chemiluminescence, 500-fold diluted OS_MYL_ and/or 2 µM H_2_O_2_ were mixed with sterile water. The mixtures were incubated at room temperature for 5 min before being subjected to the luminol-dependent chemiluminescence assay.

### 4.5. Reverse Transcription-Quantitative PCR

Total RNA extracted from suspension-cultured cells was frozen in liquid nitrogen and reverse transcription-quantitative PCR (RT-qPCR) was performed as previously described [35]. Transcript levels were analyzed using the comparative C_T_ (2^−ΔΔCt^) method with rice *ubiquitin1* (*RUBQ1*) [72,73]. Total RNA was extracted from rice leaves frozen in liquid nitrogen, and RT-qPCR was performed as previously described [74]. *OsEF1a* was used as the internal control. Primers used in this study are listed in Table S1.

### 4.6. Treaements with Wounding and Oral Secretions as Simulated Herbivory

For simulated herbivory treatments, Nipponbare, *BSR1*-OX#22, and *BSR1*-OX#42 rice plants were grown until five to seven weeks old. The youngest fully developed leaves of these plants were used. For the treatment with wounding and OS_MYL_, the leaf was mechanically wounded with a serrated tracing wheel along the midvein, immediately treated with 20 µL OS_MYL_ diluted with water (1:3; *v*/*v*), and then spread over the wounded leaf surface [74]. The untreated youngest fully developed leaves of the same age from an independent set of rice plants were used as a control.

### 4.7. Herbivore Feeding Assay

For the feeding assay on *BSR1* overexpression lines, rice plants at the vegetative stage were separately exposed to *M. loreyi* larvae. The 2nd–3rd instar larvae (5.5–7.0 mg) were attached to the youngest leaves of seven-week-old plants in a clip cage, and their individual masses were measured daily up to four days post-infestation [41]. To test the effect of momilactone B on *M. loreyi*, 50 mg of an artificial pinto-bean-based diet [75] containing momilactone B (50 mg/g) was placed into 1.5 mL tubes with nylon mesh at the top. The artificial diet containing ethanol (2.5 mL/g diet) was used as a mock control. *M. loreyi* larvae (1.3–1.8 mg, 1st instar) were placed into tubes and their mass was measured daily up to three days. Momilactone B used in this assay was purified from rice husks, as previously described [76].

### 4.8. Recovery and Analysis of Secondary Metabolites

Leaf samples for secondary metabolite analysis were collected from six-week-old rice plants treated with/without simulated herbivory as mentioned above. Phenolamides (CoP and FP), momilactone A, and momilactone B were extracted from rice leaves according to a previously published method [77]. Briefly, liquid-nitrogen-pulverized leaves (80 mg) were suspended in Extraction Buffer 1 [40% (*v*/*v*) methanol in 84 mM ammonium acetate buffer, pH 4.8]. After the addition of 5–6 ceramic beads (2.3 mm; BMS, Tokyo, Japan), samples were homogenized with FastPrep 24 (MP Biomedicals, Santa Ana, CA, USA). Suspensions were centrifuged at 16,000× g (4 °C for 15 min) and the supernatants were transferred into microtubes. Pellets were re-extracted with Extraction Buffer 2 [80% (*v*/*v*) methanol in 84 mM ammonium acetate buffer, pH 4.8], vigorously mixed at room temperature in a shaker for 10 min, and centrifuged as before. Supernatants from both extractions were combined, diluted with 84 mM ammonium acetate buffer (pH 4.8) to a final 20% (*v*/*v*) methanol concentration, and purified on a solid-phase extraction column (Bond Elut C18; Agilent Technologies, Santa Clara, CA, USA). The samples were measured on a triple quadrupole LC-MS/MS 6410 system (Agilent Technologies) equipped with a Zorbax SB-C18 column (50 × 2.1 mm inner diameter (i.d.), 1.8 mm; Agilent Technologies) as described previously [43]. The amount of each metabolite was quantified using synthetic standards (CoP and FP) or purified compounds (momilactone A and momilactone B).

The headspace VOCs emitted from rice leaves were collected from seven-week-old plants using a custom solid-phase method with a Mono Trap (GL Science Inc., Tokyo, Japan), as reported previously [48]. The VOCs were eluted from the Mono Trap and analyzed on a GC-MS system (Agilent 7890A GC, HP-5MS column, 30 m, 0.25 mm i.d., 0.25 μm film thickness; Agilent Technologies) as described previously [78]. For quantification, tetralin was used as the internal standard. Quantification was performed by comparing peak areas with standard compounds purchased from a commercial supplier (limonene, linalool, methyl salicylate, and caryophyllene; FUJIFILM Wako Pure Chemical Corporation, Osaka, Japan). Compounds with no available standards were calculated and are presented as relative peak areas normalized by the tetralin internal standard.

### 4.9. Phytohormone Measurement

Leaf samples for phytohormone analysis were collected from seven-week-old plants treated with/without simulated herbivory as mentioned above. Phytohormones (JA, jasmonoyl-_L_-isoleucine, salicylic acid, and abscisic acid) were extracted from rice leaves with ethyl acetate spiked with known amounts of deuterated internal standards and measured using an LC-MS/MS 6410 system as described previously [74]. The amount of phytohormones was calculated from the ratio of endogenous hormones to the internal standard peaks. The ethylene content was measured as previously described [42]. Ethylene released from the leaves was collected by incubating a cut leaf in a closed container for 24 h and measured using a GC-FID system (GC-2014; Shimadzu, Kyoto, Japan) equipped with a packed column (ShinCarbon ST 50/80, 2.0 m, 3 mm i.d.; Shinwa Chemical Industries Ltd., Kyoto, Japan).

### 4.10. Scanning Electron Microscopy and Histochemical Staining

Non-glandular trichomes of rice leaves (eight-week-old plants) were observed using scanning electron microscopy (Miniscope TM 3000; Hitachi High-Technologies, Tokyo, Japan), as described previously [41]. Histochemical localization of lignin in rice leaves (eight-week-old plants) was performed as described previously [79]. A saturated solution of phloroglucinol in 20% HCl was used for staining lignin. The stained cut sections of rice leaves were observed under a light microscope (BZ-X700; Keyence Corporation, Osaka, Japan).

### 4.11. Statistical Data Analyses

Statistical analyses (one-way ANOVA followed by Tukey’s HSD test) were performed using OpenStat open-source software (https://openstat.info/OpenStatMain.htm, accessed on 19 June 2020.) or a commercial version of Microsoft Excel (Student’s *t*-test). Dunnett’s test was performed using an online resource tool (http://www.gen-info.osaka-u.ac.jp/testdocs/tomocom//dunnett.html, accessed on 22 November 2022).

### 4.12. Accession Numbers

*BSR1*, Os09g0533600; *PBZ1*, Os12t0555500; *PAL1*, Os02g0627100; *DPF*, Os01g0196300; *KSL4*, Os04g0179700; *KSL7*, Os02g0570400; *KSL8*, Os11g0474800; *CPS2*, Os02g0571100; *CPS4*, Os04g0178300; *RUBQ1*, Os06g0681400; *OsDXS3*, Os07g0190000; *OsLIS*, Os02g0121700; *OsSAMT*, Os02g0719600; *OsEF1α*, Os03g0177900; *WIPI*, Os01g0132000; *PI*, Os01g0127600; *OsPR1b*, Os01g0382000.

## Data Availability

Data are contained within the article.

## Supplementary Materials

The following supporting information can be downloaded at: https://www.mdpi.com/article/10.3390/ijms241210395/s1.
