# Supplementary material for: BSR1, a Rice Receptor-like Cytoplasmic Kinase, Positively Regulates Defense Responses to Herbivory
Source: Int J Mol Sci. 2023 Jun 20;24(12):10395. doi: 10.3390/ijms241210395 (PMC10299516; doi:10.3390/ijms241210395)
Supplement: Supplementary file 1 [file ijms-24-10395-s001.zip › Supplemental Figures.pdf]

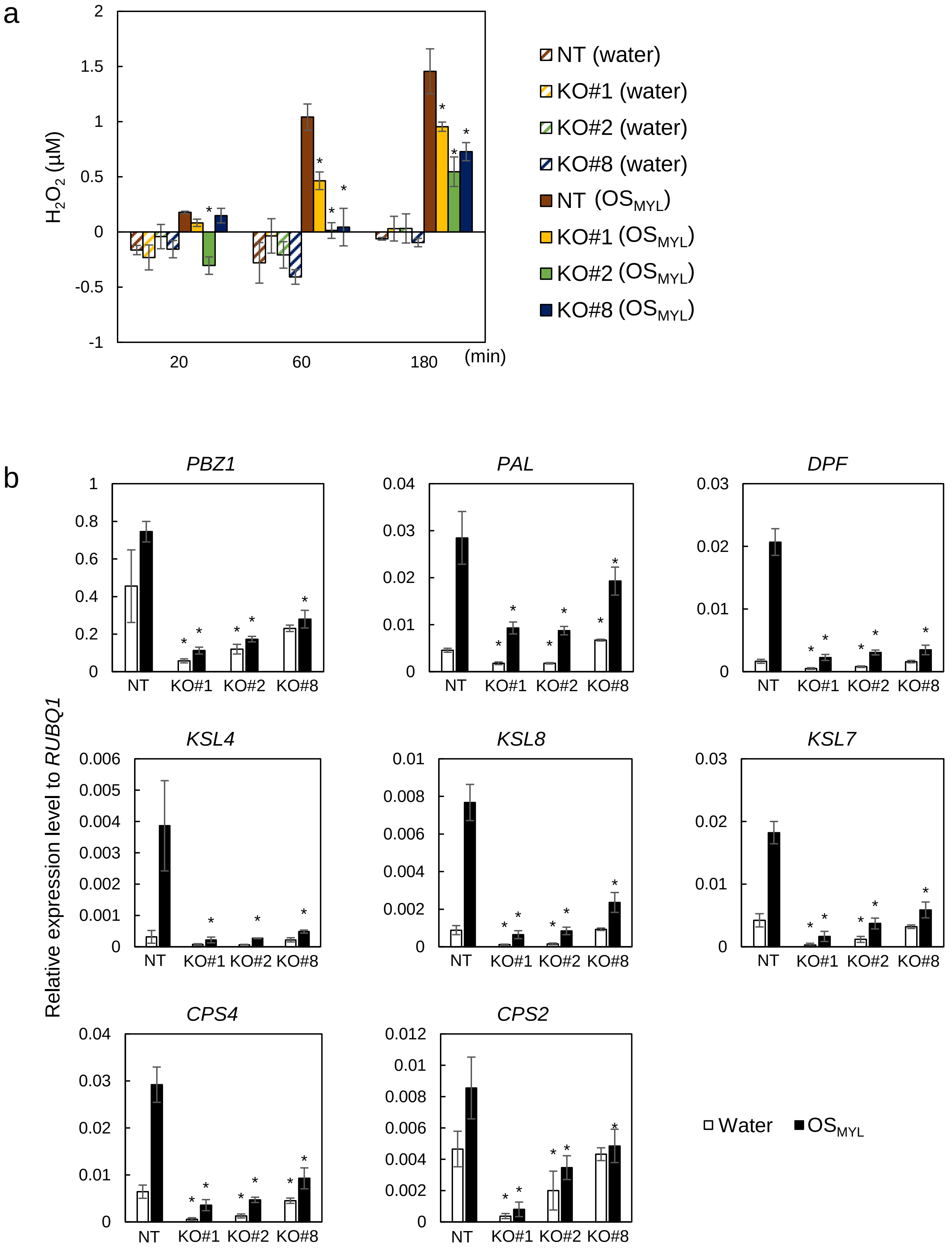

a
H_2O_2 (µM)
2
1.5
1
0.5
0
-0.5
-1
20
60
180
(min)
NT (water)
KO#1 (water)
KO#2 (water)
KO#8 (water)
NT (OS_{MYL})
KO#1 (OS_{MYL})
KO#2 (OS_{MYL})
KO#8 (OS_{MYL})
b
Relative expression level to *RUBQ1*
PBZ1
PAL
DPF
KSL4
KSL8
KSL7
CPS4
CPS2
NT
KO#1
KO#2
KO#8
Water
OS_{MYL}

Figure S1. *BSR1* knockout suppressed defense responses to *Mythimna loreyi* oral secretions. The experiments of Figure 1 were repeated to confirm reproducibility. Suspension-cultured cells were treated with OS (500-fold dilution). (a) Time course of H_2O_2 production in the cell culture. The H_2O_2 concentrations were measured before (0 min) and 20, 60, and 180 min after treatment and calculated by subtracting the value measured at 0 min from that at each time point. (b) Transcriptional activation of defense-related genes in suspension-cultured rice cells. The *PBZ1*, *PAL1*, *DPF, KSL4, KSL8, KSL7, CPS2, and CPS4* transcript levels 3 h after treatment with OS were normalized against the *RUBQ1* internal control levels. Asterisks indicate significant differences between the values of NT and those of the other lines (Dunnett's test; $*P < 0.05$). Values are presented as the mean ± standard deviation of three biological replicates. Water, treated with sterile water; OS_{MYL}, treated with *Mythimna loreyi* oral secretions; KO, *BSR1*-knockout line; NT, non-transgenic line (Nipponbare).

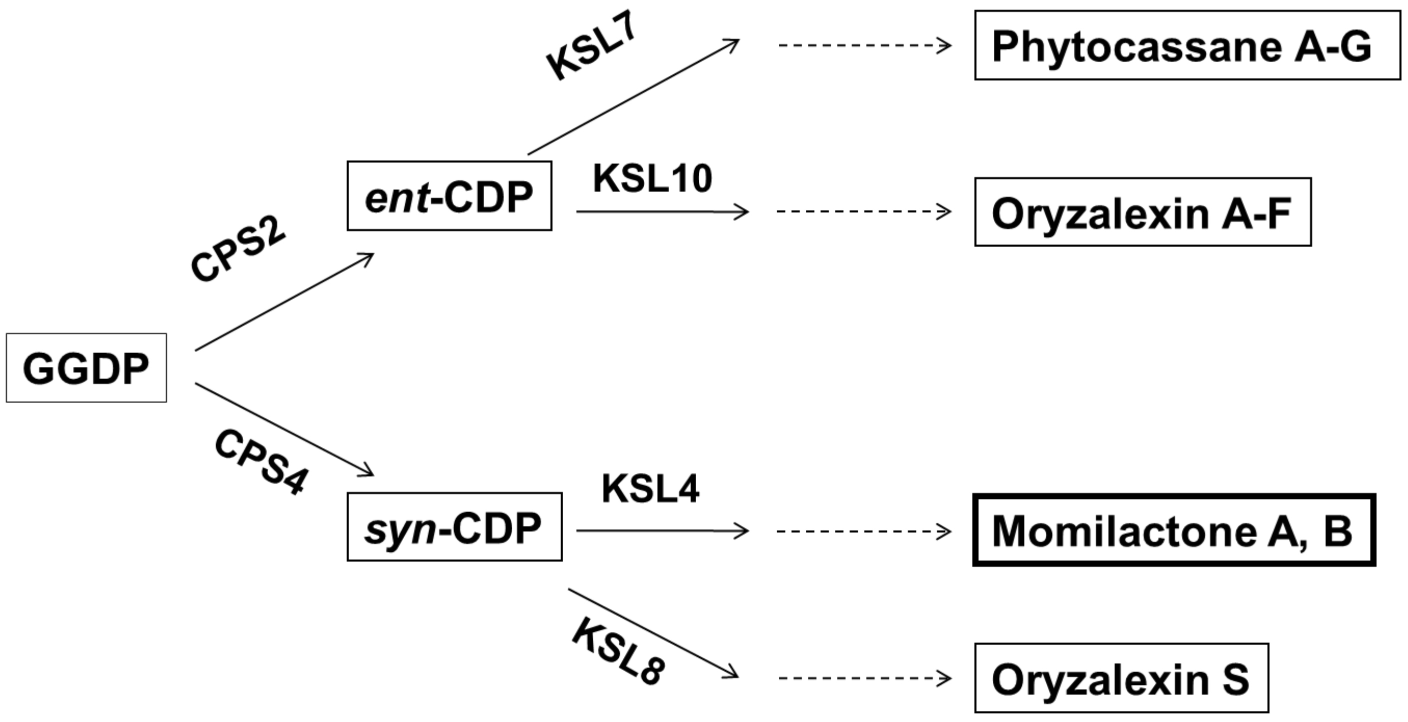

Figure S2. Schematic biosynthetic pathway of diterpenoid phytoalexins in rice. Solid arrows, reactions catalyzed by the indicated enzyme; dashed arrows, multiple reactions; GGDP, geranylgeranyl diphosphate; CDP, copalyl diphosphate.

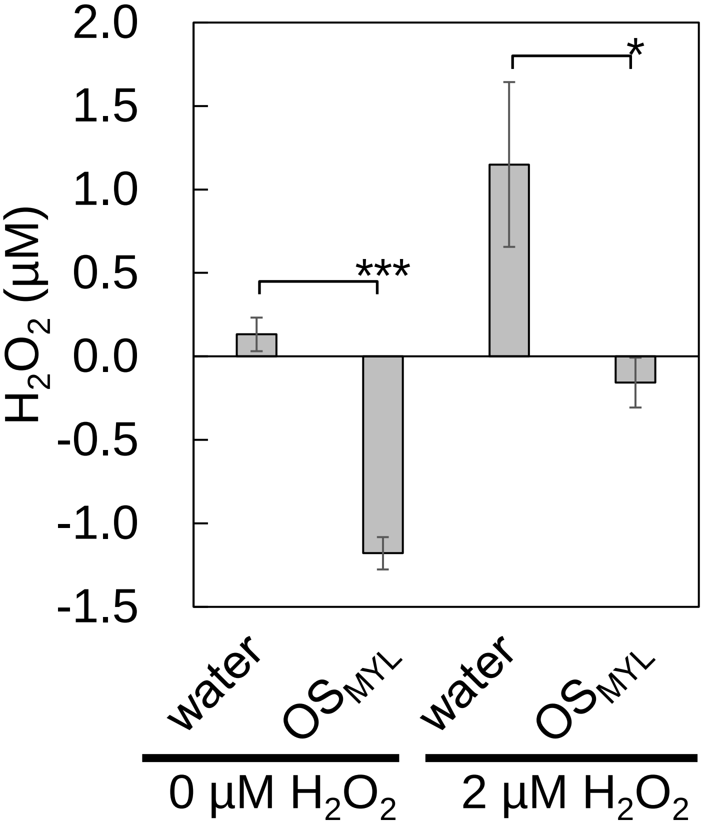

Figure S3. OS_{MYL} inhibited the luminol chemiluminescence assay, regardless of the presence of H_2O_2. Sterile water or 500-fold diluted OS_{MYL} were mixed with 0 µM H_2O_2 (water) or 2 µM H_2O_2 and then subjected to the luminol-dependent chemiluminescence assay. Asterisks indicate significant differences between indicated values (*t*-test; $*P < 0.05$ and $***P < 0.001$). Values are presented as the mean ± standard deviation of three experimental replicates.

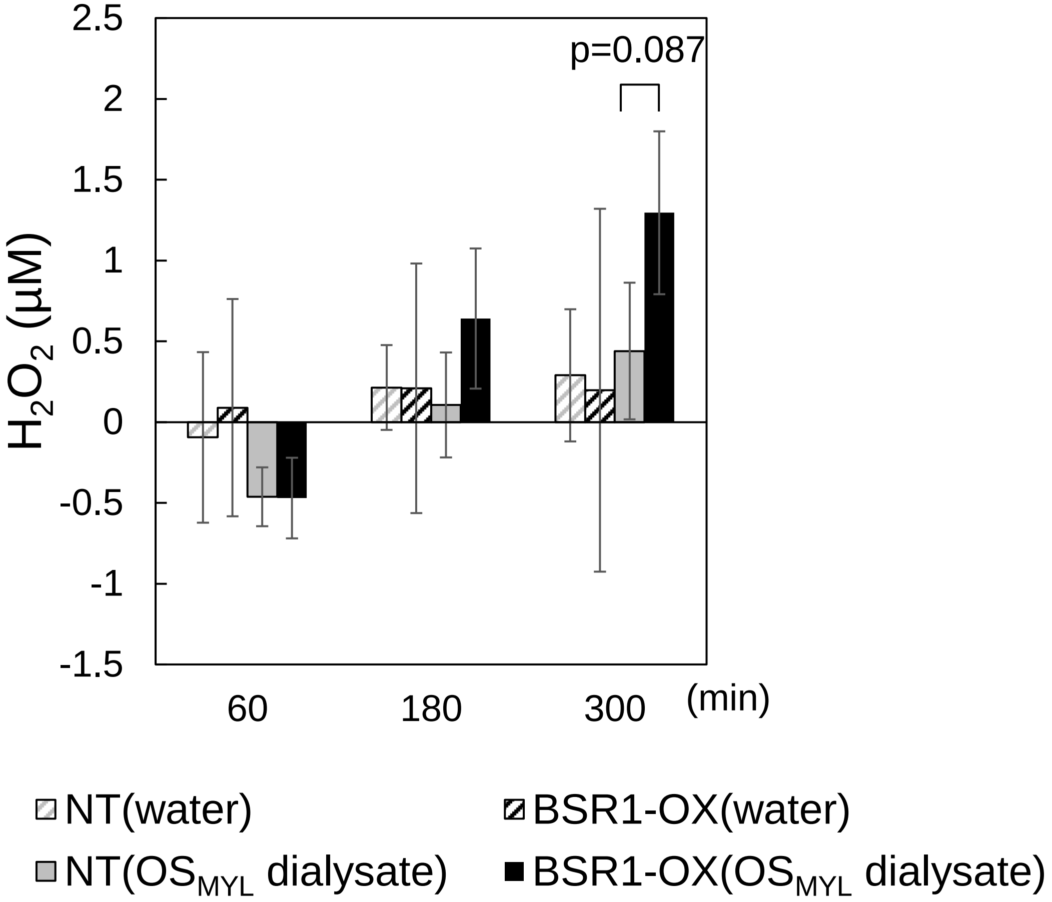

Figure S4. *BSR1* overexpression enhanced oral secretion-triggered ROS burst. The experiment of Figure 2 was repeated to confirm reproducibility. Leaf strips were treated with water or dialyzed OS_{MYL}. The H_2O_2 concentrations were measured before (0 min) and 60, 180, and 300 min after treatment and calculated by subtracting the value measured at 0 min from that at each time point. Asterisks indicate significant differences between the values of NT and those of the other lines (*t*-test; $*P < 0.05$, $**P < 0.01$, and $***P < 0.001$). Values are presented as the mean ± standard deviation of three biological replicates. Water, treated with sterile water; OS_{MYL} dialysate, treated with dialyzed oral secretion; NT, non-transgenic line (Nipponbare) ; BSR1-OX, *BSR1*-overexpressing line.

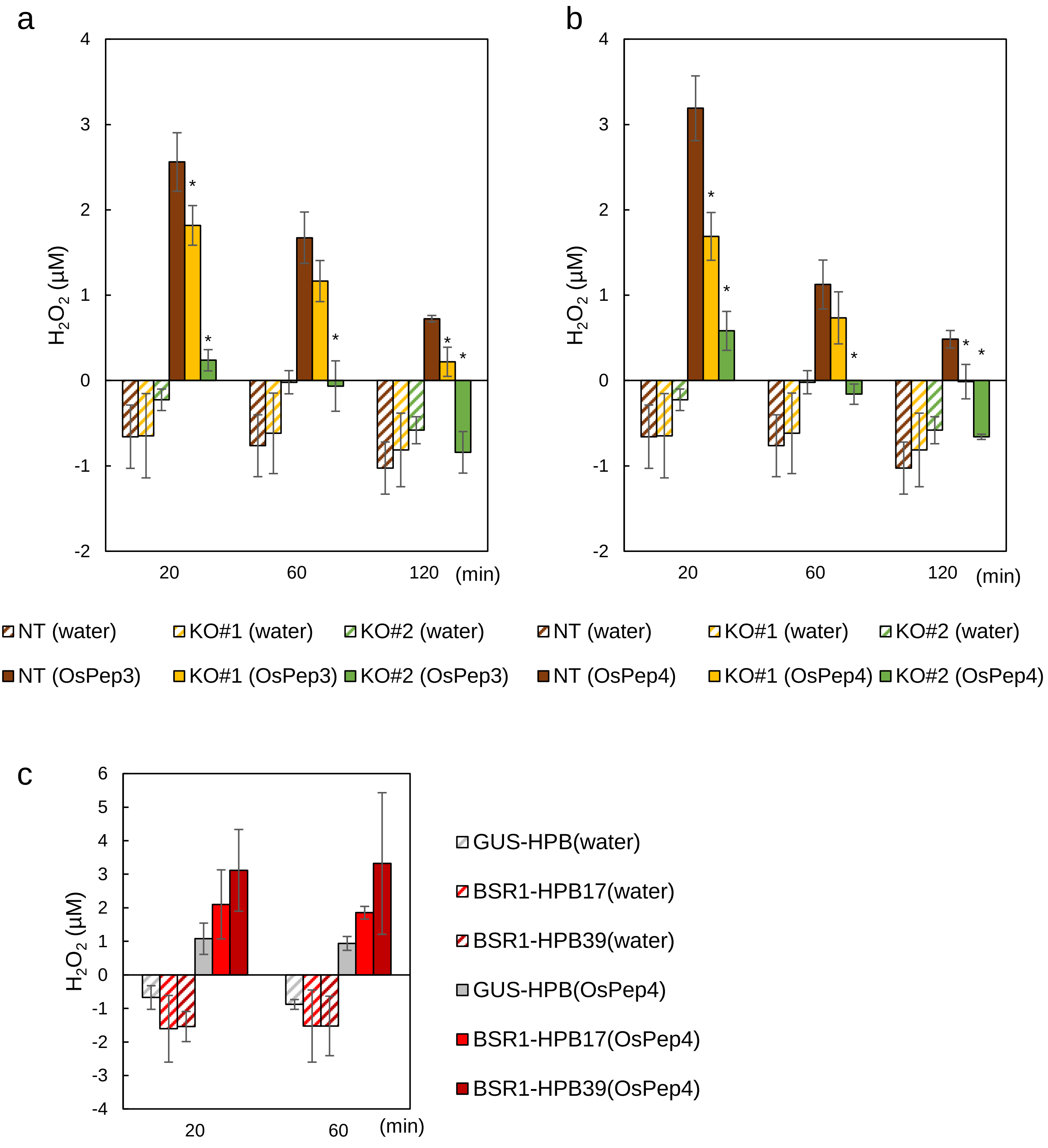

Figure S5. BSR1 mediates OsPep3- and OsPep4-triggered ROS burst. The experiments of Figure 3 were repeated to confirm reproducibility. (a, b) *BSR1*-KO suspension-cultured cells were treated with OsPep3 (a) and OsPep4 (b). The H_2O_2 concentrations were measured before (0 min) and 20, 60, and 120 min after treatment and calculated by subtracting the value measured at 0 min from that at each time point. Asterisks indicate significant differences between the values of NT and those of KO lines under the same treatment conditions (Dunnett's test; $^*P < 0.05$). (c) *BSR1*-OX suspension-cultured cells were treated with OsPep4, and H_2O_2 concentrations were measured before and 20 and 60 min after treatment. Values are presented as the mean ± standard deviation of three biological replicates. Water, treated with sterile water; NT, non-transgenic line (Nipponbare); KO, *BSR1*-knockout line.

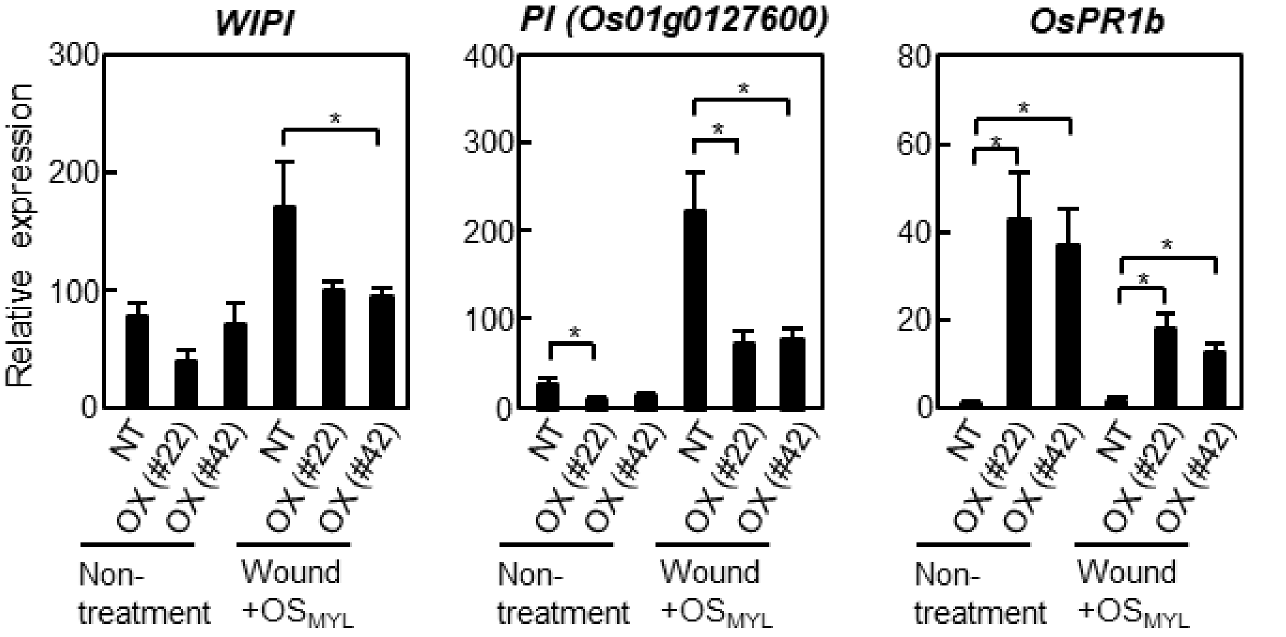

Figure S6. Expression of defense-related genes in *BSR1*-OX. Rice leaves were collected at 1 h after the treatment. Data are shown as mean ± SE (independent biological replicates, n=6). Statistical differences were analyzed by Dunnett's test (*$P < 0.05$). NT, non-transgenic line (Nipponbare); OX (#22) and OX (#42), BSR1-overexpressing lines #22 and #42.

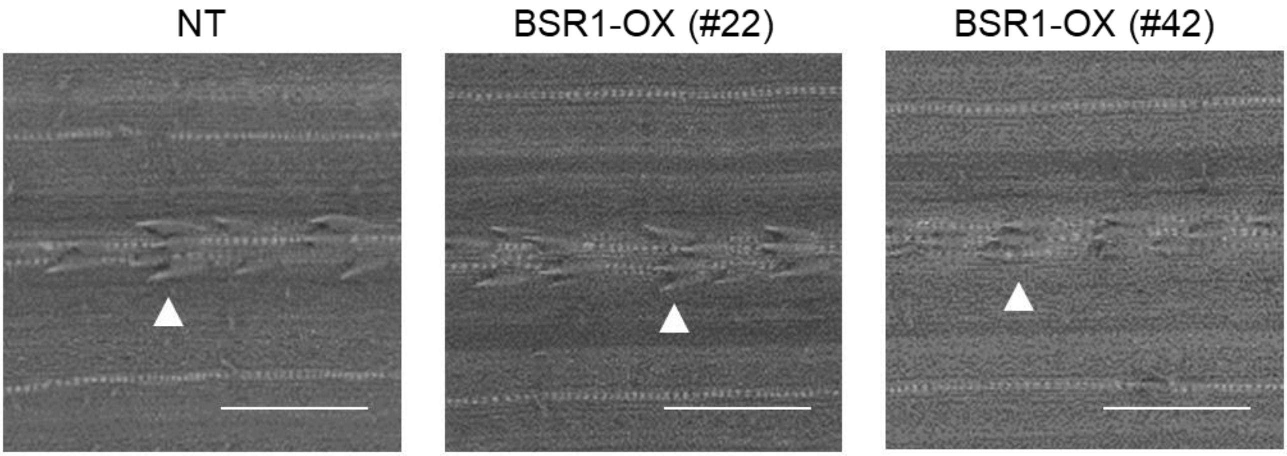

Figure S7. Surface scanning electron microscope (SEM) images of rice leaves. Fresh rice leaves were fixed on a stage and observed by SEM. The arrows indicate non-glandular silicified trichomes. Bar = 1.0 mm. NT, non-transgenic line (Nipponbare); BSR1-OX (#22) and BSR1-OX (#42), BSR1-overexpressing lines #22 and #42.

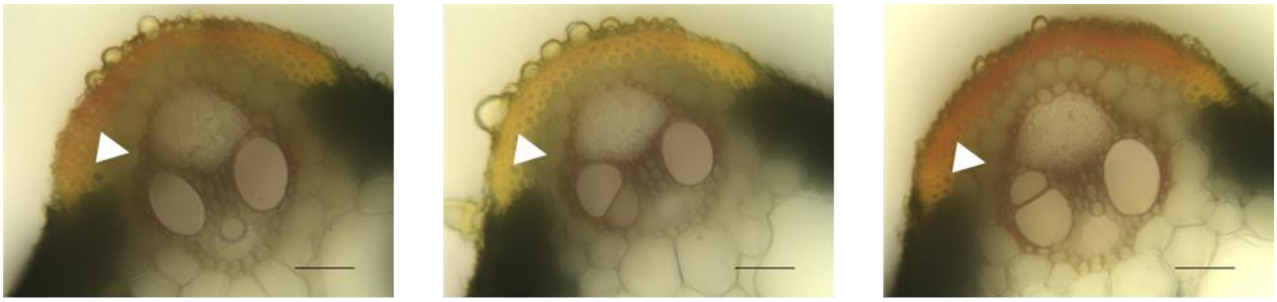

Figure S8. Lignin accumulation in non-transgenic and *BSR1*-OX rice leaves. Histochemical staining with phloroglucinol shows lignin accumulation (a red-violet color indicated by arrow). Bar = 50 mm. NT, non-transgenic line (Nipponbare); BSR1-OX (#22) and BSR1-OX (#42), BSR1-overexpressing lines #22 and #42.

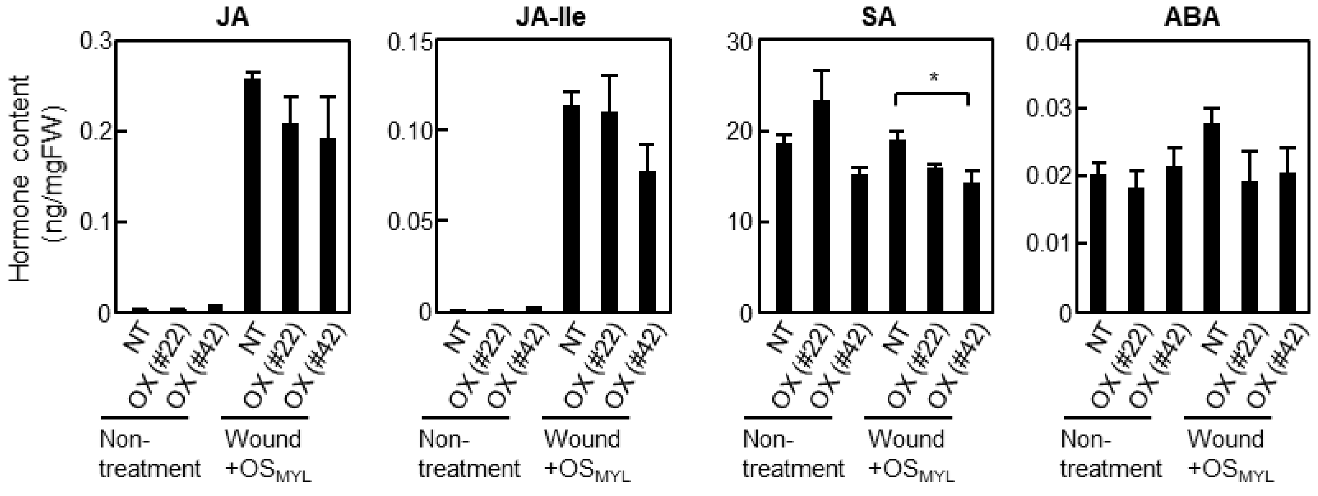

Figure S9. Phytohormone production in *BSR1*-OX. Rice leaves were collected at 1 h after the treatment. Data are shown as mean ± SE (independent biological replicates, n=4). Statistical differences were analyzed by Dunnett's test (*P < 0.05). JA, jasmonic acid; JA-Ile, jasmonoyl-L-isoleucine; SA, salicylic acid; ABA, abscisic acid; NT, non-transgenic line (Nipponbare); OX (#22) and OX (#42), BSR1-overexpressing lines #22 and #42.

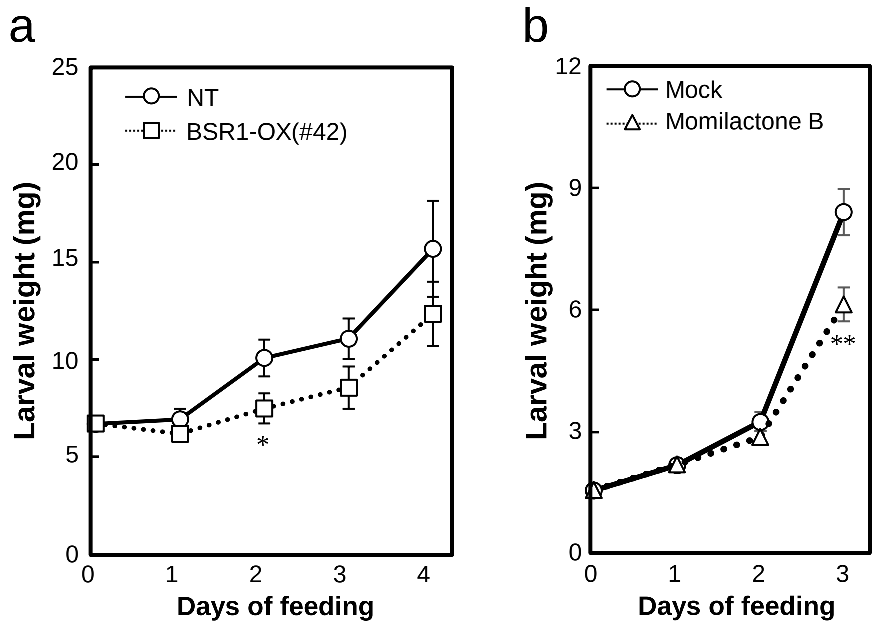

Figure S10. Larval growth performance of *Mythimna loreyi.* (a) *BSR1* overexpression in rice decreases the performance of *M. loreyi* larvae. Since we had observed similar differences in two *BSR1*-OX lines, as shown in main Figure 7a, we tested one *BSR1*-OX line in the supplementary assay. Larval masses are shown as mean ± SE (independent biological replicates, n=13-16). Statistical differences were analyzed in each time-point by Student's *t*-test (**P* < 0.05). (b) Biological activity of momilactone B against *M. loreyi*. *M. loreyi* larvae were individually fed on mock or momilactone B-containing artificial diet. Data are shown as mean ± SE (independent biological replicates, n= 32-33). Statistical differences were analyzed in each time-point by Student's *t*-test (***P*< 0.01).
