# Supplementary material for: BSR1, a Rice Receptor-like Cytoplasmic Kinase, Positively Regulates Defense Responses to Herbivory
Source: Int J Mol Sci. 2023 Jun 20;24(12):10395. doi: 10.3390/ijms241210395 (PMC10299516; doi:10.3390/ijms241210395)
Supplement: Supplementary file 1 [file ijms-24-10395-s001.zip › Supplemental Table.pdf]

Supplemental Table S1. Primers used for RT-qPCR in this work.

Genes	primer 1	primer 2
PBZ1	CCGGCTTGGTCGACGACATT	CCGACTTTAGGACATGACTT
PAL1	GCTATCAACGAAGGCAAGCAC	GCCTCCACACTCCACTGTTATTC
DPF	CGTGCAAACCTAACATTACA	GGCACCTCCCTTTTTCTTCTT
KSL4	GTATTTCATGGGACAAAATCTCTGG	CCATCCTTGCATTCCCTCTC
KSL7	TCTACTACCAGACCGACGGATTC	GAGTTGAAGTGGCTCGTTGATG
KSL8	ATTAATTCCTACGTCTTGTTGCTTGGC	ATATGCGCGGGTTTTCTTCCTAGTTTTAG
CPS2	CGTGAAGCGTGGAAGCAGT	GCGTAGTCTGCACTGTTCTT
CPS4	TGACGAGGCTGGGCATATC	TCTGGAGTCCAGTTCCTGAAA
RUBQ1	GGAGCTGCTGCTGTTCTAGG	TTCAGACACCATCAAACCAGA
OsDXS3	GGGGGAGGTTCCAGTAAGAA	TCATTTTGCATTTGGAAGCA
OsLIS	TTTGATGGATTCATGACAGAGA	CAACAAACTCTGCTGCATTTT
OsSAMT	CTCATCGCTCGTCATTTCGG	ACACACTGGCACGCTACTTA
OsEF1a	CTGCCACACCTCCCACATTGC	CCGCACGGCAAAACGACCA
WIPI	ATAATGCATGAACGCGAGCC	TCCTTCACTCAGACAACGGC
PI	AAGTGTCACAACCAGCCAGC	TATGTCACTGCTACTCGGCA
OsPR1b	ACGGGCGTACGTACTGGCTA	CTCGGTATGGACCGTGAAG
